# Elucidation of repeat motifs R1‐ and R2‐related *TRIOBP* variants in autosomal recessive nonsyndromic hearing loss DFNB28 among indigenous South African individuals

**DOI:** 10.1002/mgg3.2015

**Published:** 2022-08-27

**Authors:** Rosemary Ida Kabahuma, Wolf‐Dieter Schubert, Christiaan Labuschagne, Denise Yan, Michael Sean Pepper, Xue‐Zhong Liu

**Affiliations:** ^1^ Department of Otorhinolaryngology University of Pretoria Pretoria South Africa; ^2^ Departments of Biochemistry, Genetics and Microbiology, Faculty of Natural and Agricultural Sciences University of Pretoria Pretoria South Africa; ^3^ Inqaba Biotechnology Pretoria South Africa; ^4^ Department of Otolaryngology University of Miami Miller School of Medicine Miami Florida USA; ^5^ Institute for Cellular and Molecular Medicine, Department of Immunology and SAMRC Extramural Unit for Stem Cell Research and Therapy, Faculty of Health Sciences University of Pretoria Pretoria South Africa

**Keywords:** autosomal recessive nonsyndromic hearing loss, DFNB28, indigenous South Africans, TRIOBP R1 and R2 repeat motifs

## Abstract

**Background:**

DFNB28, a recessively inherited nonsyndromic form of deafness in humans, is caused by mutations in the *TRIOBP* gene (MIM #609761) on chromosome 22q13. Its protein TRIOBP helps to tightly bundle F‐actin filaments, forming a rootlet that penetrates through the cuticular plate into the cochlear hair cell body. Repeat motifs R1 and R2, located in exon 7 of the TRIOBP‐5 isoform, are the actin‐binding domains. Deletion of both repeat motifs R1 and R2 results in complete disruption of both actin‐binding and bundling activities, whereas deletion of the R2 motif alone retains F‐actin bundling ability in stereocilia rootlets.

**Methods:**

Target sequencing, using a custom capture panel of 180 known and candidate genes associated with sensorineural hearing loss, bioinformatics processing, and data analysis were performed. Genesis 2.0 was used for variant filtering based on quality/score read depth and minor allele frequency (MAF) thresholds of 0.005 for recessive NSHL, as reported in population‐based sequencing databases. All variants were reclassified based on the American College of Medical Genetics and Genomics (ACMG) and Association for Molecular Pathology (AMP) guidelines together with other variant interpretation guidelines for genetic hearing loss . Candidate variants were confirmed via Sanger sequencing according to standard protocols, using the ABIPRISM 3730 DNA Analyzer. DNA sequence analysis was performed with DNASTAR Lasergene software.

**Results:**

Candidate *TRIOBP* variants identified among 94 indigenous sub‐Saharan African individuals were characterized through segregation analysis. Family TS005 carrying variants c.572delC, p.Pro191Argfs*50, and c.3510_3513dupTGCA, p.Pro1172Cysfs*13, demonstrated perfect cosegregation with the deafness phenotype. On the other hand, variants c.505C > A p.Asp168Glu and c.3636 T > A p.Leu1212Gln in the same family did not segregate with deafness and we have classified these variants as benign. A control family, TS067, carrying variants c.2532G > T p.Leu844Arg, c.2590C > A p.Asn867Lys, c.3484C > T p.Pro1161Leu, and c.3621 T > C p.Phe1187Leu demonstrated no cosegregation allowing us to classify these variants as benign. Together with published TRIOBP variants, the results showed that genotypes combining two truncating TRIOBP variants affecting repeat motifs R1 and R2 or R2 alone lead to a deafness phenotype, while a truncating variant affecting repeat motifs R1 and R2 or R2 alone combined with a missense variant does not. Homozygous truncating variants affecting repeat motif R2 cosegregate with the deafness phenotype.

**Conclusion:**

While a single intact R1 motif may be adequate for actin‐binding and bundling in the stereocilia of cochlear hair cells, our findings indicate that a truncated R2 motif *in cis* seems to be incompatible with normal hearing, either by interfering with the function of an intact R1 motif or through another as yet unknown mechanism. Our study also suggests that most heterozygous missense variants involving exon 7 are likely to be tolerated.

## Graphical abstract

A South African family with confirmed DFNB28, an autosomal recessive nonsyndromic hearing loss (ARNSHL) due to compound heterozygous mutations in TRIOBP, c.572del (p.Pro191Argfs*50) which ablates both repeat motifs R1 and R2, and c.3510_3513dupTGCA (p.Pro1172Cysfs*13) which truncates the protein inside repeat motif R2. The characterization of these mutations through segregation analysis, in comparison to missense TRIOBP mutations of unknown significance in another South African family from the same geographical region as a control, was undertaken. The families provide an excellent platform for the elucidation of compound heterozygous TRIOBP mutations and confirm the importance of the R1 actin binding/bundling repeat motif in the pathophysiology of DFNB28 in this sub‐Saharan African population.

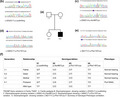

## 1 INTRODUCTION

DFNB28, a recessively inherited nonsyndromic form of deafness in humans, is caused by mutations in the gene *TRIOBP* (MIM #609761) (Riazuddin et al., 2006; Shahin et al., 2006). Its product, TRIOBP, is an F‐actin bundling protein that regulates actin cytoskeleton organization, cell growth, and cell migration. Transmission electron microscopy (TEM) shows that the arrangement of the actin within the tapered end of the stereocilia provides the resilience required for pivoting the stereocilia when deflected in response to sound stimulation (Furness et al., 2008; Kitajiri et al., 2010; Tilney et al., 1980).

Different isoforms of TRIOBP produced by the use of at least two known alternate promoters are reported (Kitajiri et al., 2010). They are grouped into three classes. Transcript TRIOBP‐1 contains C‐terminal domains encoded by exons 11–24 but excludes N‐terminal domains including repeat motifs R1 and R2 (Seipel et al., 2001). Transcript TRIOBP‐4 is encoded by exons 2–7 (previously exons 1–6), producing a protein with the internal repeat motifs, R1 and R2, but lacking the C‐terminal domains. The longest transcript, TRIOBP‐5, contains both the N‐terminal internal repeat motifs R1 and R2 and the C‐terminal domains. While TRIOBP‐1 is ubiquitously expressed, TRIOBP‐4 and ‐5 are predominantly expressed in the inner ear and in the eye. Murine TRIOBP‐5 locates at the base of the rootlets of the cochlear hair cell stereocilia by postnatal days 1 and 2, before extending into the cuticular plate along the mature rootlet (Kitajiri et al., 2010). TRIOBP‐4, by contrast, binds to dense bundles of F‐actin filaments (Kitajiri et al., 2010).

TRIOBP coordinates the tight packing of F‐actin filaments into bundles to create rootlets penetrating through the cuticular plate into the cell body (Furness et al., 2008; Kitajiri et al., 2010; Tilney et al., 1980). Knockout mouse equivalents of human DFNB28 demonstrated that although TRIOBP‐4/TRIOBP‐5‐null mice develop normal length stereocilia, the rootlets of the hair cells do not develop, causing fragile, abnormally flexible stereocilia (Kitajiri et al., 2010).

In TRIOBP‐5, the repeat motifs R1 and R2 encoded by exon 7 are essential for actin binding (Bao et al., 2013). Deletion of both R1 and R2 abolishes actin‐binding and bundling by TRIOBP‐4 and ‐5 entirely (Bao et al., 2013). Deletion of motif R2 alone retained F‐actin bundling and TRIOBP localization to the cellular actin cytoskeleton, whereas R1‐deficient TRIOBP‐4 and TRIOBP‐5 did not colocalize to actin filaments and formed thin F‐actin bundles. Overall, R1 was thus inferred to be the major actin‐binding/bundling domain, whereas R2 has a lesser role.

We previously reported *TRIOBP* mutations in a deaf indigenous South African individual, classified according to the recommendations of the Human Genome Variant Society (HGVS) and the American College of Medical Genetics (ACMG) as likely pathogenic (Yan et al., 2016). We subsequently further characterized these mutations and hereby report our findings. The families provide an excellent platform for the elucidation of compound heterozygous *TRIOBP* variants and confirm the importance of the R1 and R2 actin‐binding/bundling repeat motifs in the pathophysiology of DFNB28 in this sub‐Saharan African population.

## 2 METHODS

This study was approved by the Research Ethics Committee of the Faculty of Health Sciences, University of Pretoria, South Africa, Ethics Approval number 395/2014, and the Institutional Review Board of the University of Miami, Miller School of Medicine (USA). Signed informed consent was obtained from each participant or, in the case of a minor, from the parents.

### 2.1 Families and clinical evaluation

The families with recessive HL described in this study are part of the 94 indigenous sub‐Saharan African individuals who tested negative for the common deafness‐related mutations previously reported (Kabahuma et al., 2011; Yan et al., 2016).

### 2.2 Target enrichment sequencing and bioinformatics analysis

Target sequencing, using a custom capture panel of 180 known and candidate genes associated with sensorineural hearing loss, bioinformatics processing, and data analysis were performed as previously described (Kabahuma et al., 2021; Yan et al., 2016). Genesis 2.0 (https://www.genesis‐app.com/) was used for variant filtering based on quality/score read depth and the minor allele frequency (MAF) thresholds of 0.005 for recessive NSHL, as reported in population‐based sequencing databases. All variants were reclassified based on the American College of Medical Genetics and Genomics (ACMG) and Association for Molecular Pathology (AMP) guidelines (Richards et al., 2015), together with the variant interpretation guidelines for genetic hearing loss as published by Oza et al. (2018).

### 2.3 Sanger sequencing

Candidate variants were confirmed via Sanger sequencing according to standard protocols. Primer3, v. 0.4.0 (http://primer3.ut.ee) was used for primer design. PCR products were purified with Qiagen Qiaquick purification kit and bidirectionally sequenced using the ABI PRISM Big Dye Terminator Cycle Sequencing V3.1 Ready Reaction Kit and passed on to the ABIPRISM 3730 DNA Analyzer (Applied Biosystems). DNA sequence analysis was performed with DNASTAR Lasergene software. NCBI Reference Sequence: NC_000022.11 was used.

## 3 RESULTS

**FIGURE 1:**
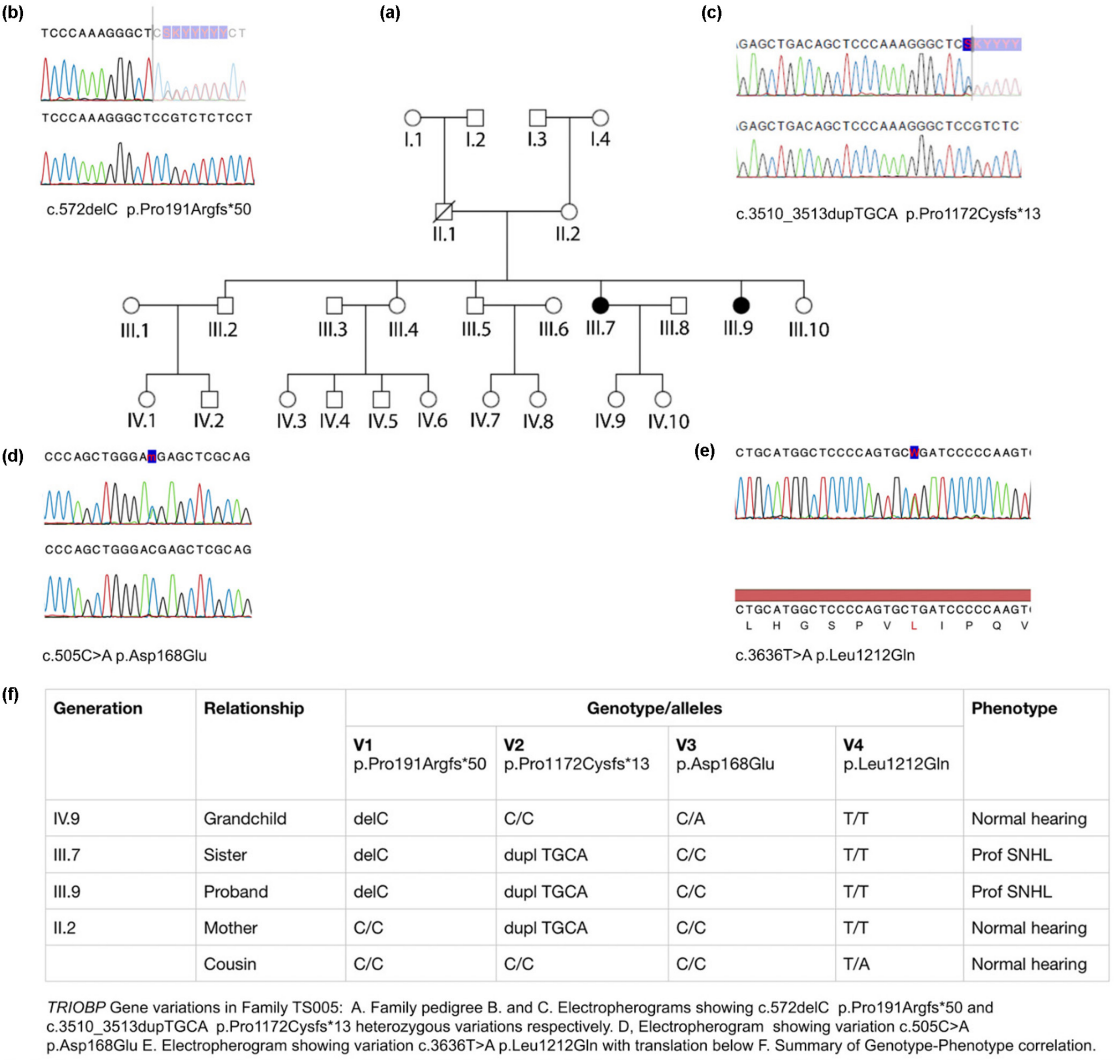
Segregation analysis of South African family TS005. NCBI Reference Sequence: NC_000022.11 used

**FIGURE 2:**
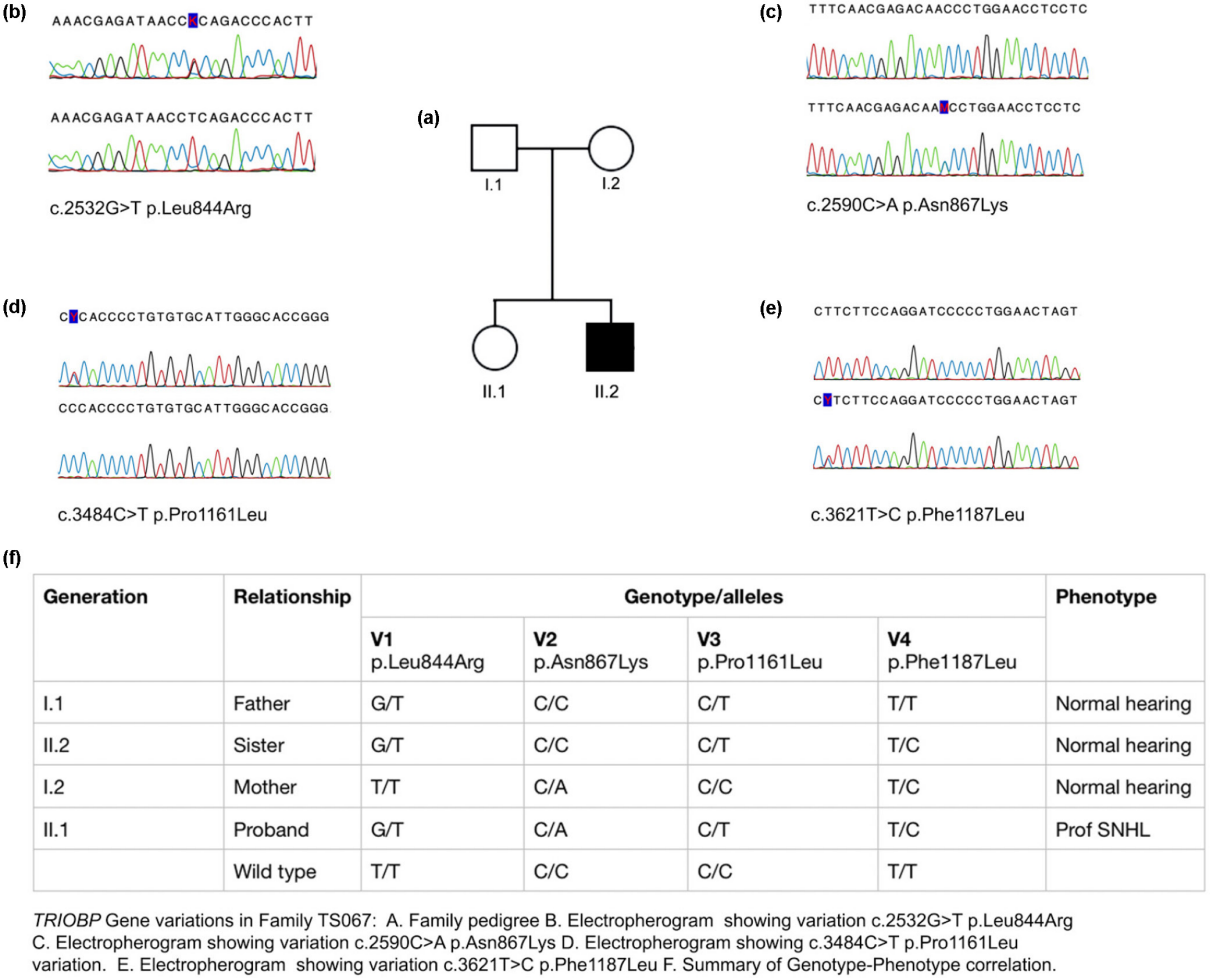
Segregation analysis of South African family TS067. NCBI Reference Sequence: NC_000022.11 used

In members of an indigenous African family TS005 from the Limpopo Province of South Africa, one of six siblings born to normally hearing parents (III.9) presenting with prelingual, sensorineural, bilateral, symmetric profound hearing loss, was initially screened and found to be negative for mutations in *GJB2*, the *GJB6*‐D13S 1830 mutation, and four mitochondrial mutations A1555G, A7445G, A3243G, and A7511C (Figure 1). We identified two *TRIOBP* frameshift variants in this individual, heterozygous c.572delC p.Pro191Argfs*50, as well as a heterozygous insertion, c.3510_3513dupTGCA, p.Pro1172Cysfs*13 which demonstrated perfect segregation with the deafness phenotype (Figure 1) on segregation analysis (Figure 1). We also identified two missense variants, c.505C > A p.Asp168Glu and c.3636 T > A p.Leu1212Gln in the same family which did not segregate with the deafness phenotype. We have classified these variants as benign. Family TS067 in whom compound heterozygous TRIOBP variants of uncertain significance had been identified was used as a control (Figure 2). The proband, born to normal hearing parents, presented with prelingual nonsyndromic hearing loss. His sister, a slow learner, has normal hearing. Compound heterozygous missense variants, c.2532G > T p.Leu844Arg, c.2590C > A p.Asn867Lys, c.3484C > T p.Pro1161Leu, and c.3621 T > C p.Phe1187Leu, were identified in this family (Figure 2). On segregation analysis, none of the family genotypes segregated with the deafness phenotype. We have also classified these variants as benign.

### 3.1 Analysis of the location of published variants in relation to TRIOBP repeat motifs R1 and R2

**FIGURE 3:**
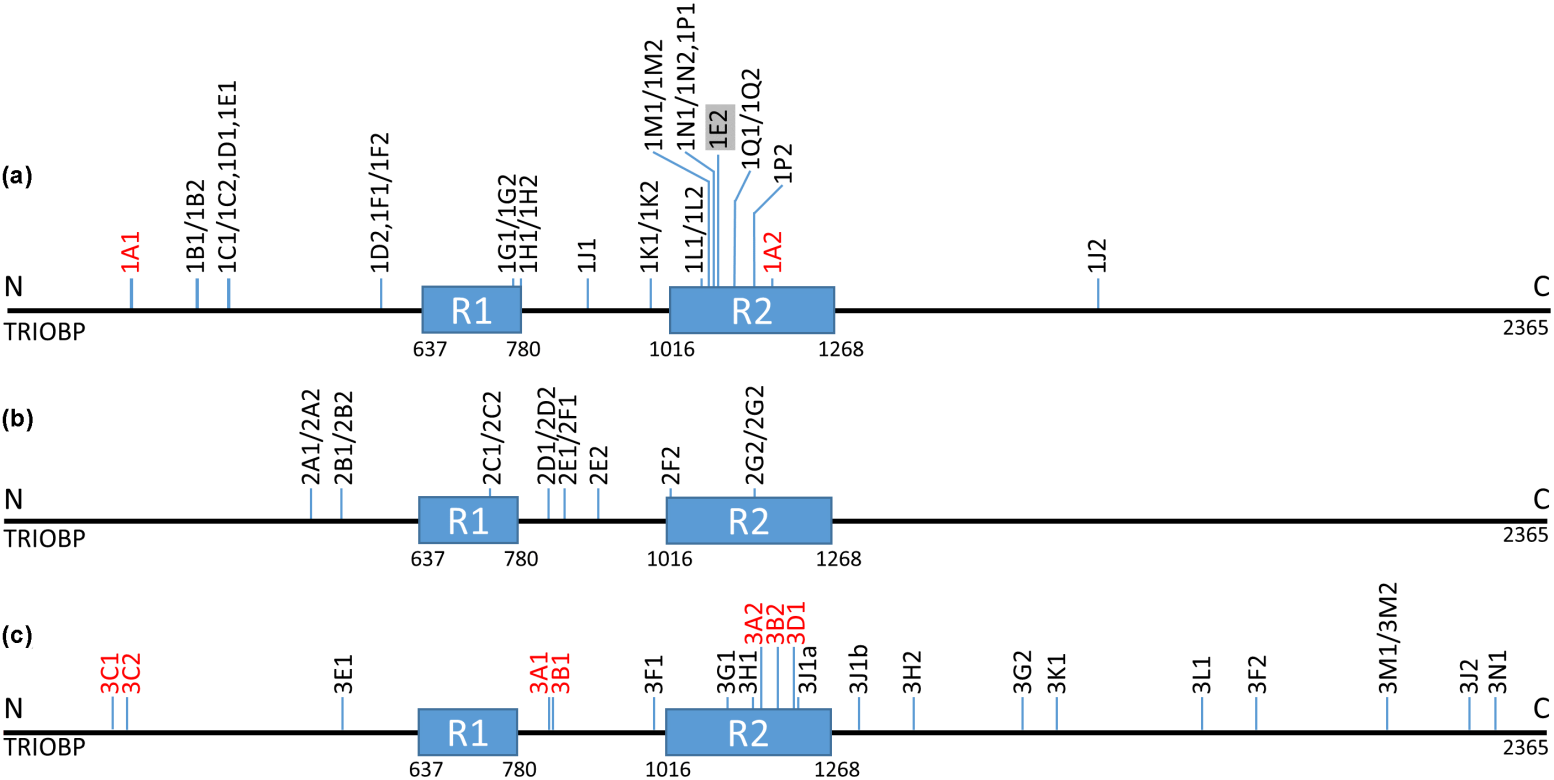
Sites of reported TRIOBP frameshift/deletion and point mutation variants relative to R1 and R2 repeat motifs. Panels A, B, and C refer to reported coded variants listed in Tables 1, 2, 3, respectively (detailed data supplementary Tables S1–S3). All variants in panels A and B are truncating except 1E2. All panel C variants are nontruncating except 3C2 and 3D1. Variants introduced in this study are marked in red. NCBI Reference Sequence: NC_000022.11 used

All variants in panel A of Figure 3 are either frameshift truncating or truncating, except for variant code 1E2, which is a missense variant, p.Gly1019Arg. Of the 15 individuals represented, the genotypes of 10 individuals are homozygous and five are heterozygous. Except for variant p.Gly1672* (allele code 1 J2, exon 9) and variant p.Pro191Argfs*50 (allele code 1A1, exon 6), all variants are located in exon 7. Note that all the variants, except p.Gly1019Arg (allele code 1E2) and p.Gly1672* (allele code 1 J2), truncate the protein causing partial loss of R2 only or of R1 and R2.

All 14 variants represented in panel B (Figure 3) are truncating, with one also causing a frameshift. Five of the seven individuals present as homozygous while the remaining two individuals are heterozygous. All variants are encoded by exon 7 and truncate the protein causing (partial) loss of repeat motifs R2 only or of R1 and R2.

Panel C (Figure 3) represents two truncating variants and 20 missense variants among 13 individuals. The variants are heterozygous in eight individuals, while the second allele is unknown in five individuals. The variants are widely distributed, from exon 6 to exon 22. Six variants are located in exon 7, including the control variants identified in the current study. Two control variants from the current study are encoded by exon 6. Three published variants map to exon 9, and one variant each to exons 14, 16, 18, 21, and 22. Allele codes 3C2, p.Pro191Argfs*50, and 3D1, p.Arg1117*, are the only truncating variants causing most or all of the repeat motifs R1 and R2 to be lost.

All the variants and genotypes represented in panels A and B cause a deaf phenotype, except for two individuals with allele codes 1E1/1E2 and 1J1/1J2 whose genotype remains to be elucidated. Note that allele code 1E2, p.Gly1019Arg, is a missense variant that does not truncate the protein, does not cause the loss of repeat motifs R1 and R2, and hence groups with the other missense variants in panel C. The genotype of this individual therefore should not lead to a deaf phenotype. Allele 1J2 on the other hand is an outlier, located far downstream of repeat motifs R1 and R2. We propose that despite it being a truncating variant, it is unlikely to affect the actin‐binding and bundling activities of the repeat motifs R1 and R2. Correspondingly, this genotype is unlikely to lead to a deaf phenotype.

Variants in panel C were classified as of uncertain significance in the various publications. Our control variants are also located in this panel. We demonstrated from our family studies that the control variants from both families TS005 and TS067 are shared by normal‐hearing individuals and deaf probands. They, therefore, do not segregate with the deaf phenotype. The deaf proband in Family TS067 also carries variants in two other known deafness genes which are currently under investigation. Because panel C variants, except p.Pro191Argfs*50 (allele code 3C2) and p.Arg1117* (allele code 3D1), do not truncate the protein, they are unlikely to affect the actin‐binding and bundling activity of the TROBP R1 and R2 repeat motifs. Furthermore, the second allele could not be identified in five individuals. Although both p.Pro191Argfs*50 and p.Arg1117* have been shown to cosegregate with the deafness phenotype, p.Pro191Argfs*50 in combination with p.Pro1172Cysfs*13 (allele codes 1A1/1A2) and p.Arg1117* in the homozygous state (allele codes 1Q1/1Q2) in panel A above, it is unlikely that the combination of p.Arg1117* with a missense variant in this individual (allele codes 3D1/3D2) would lead to deafness. We have shown that the combination of the truncating variant p.Pro191Argfs*50 with a missense variant p.Asp168Glu does not cause a deaf phenotype (Figure 1). We thus infer that the genotypes in panel C generally do not lead to a deaf phenotype.

## 4 DISCUSSION

We present an indigenous South African family, TS005, with confirmed DFNB28 which presents an opportunity for the further elucidation of TRIOBP variants in the pathogenesis of DFNB28. The mother is the carrier of the c.3510_3513dupTGCA variant while it is presumed that the deceased father was the carrier of the c.572delC deletion. Both parents had normal hearing. Whereas the first variant (V1) c.572del p.Pro191Argfs*50, allele code 1A1, ablates both repeat motifs R1 and R2 *in cis*, the second variant c.3510_3513dup p.Pro1172Cysfs*13, allele code 1A2, spares the R1 repeat motif but truncates the last third of the R2 repeat motif. The individuals in family TS005 with both mutations are deaf yet carry one intact R1 motif and one truncated R2 motif *in cis*.

Functional studies indicated that deletion of both repeat motifs R1 and R2 results in complete disruption of both actin‐binding and bundling activities, while deletion of the R2 motif alone retains the F‐actin bundling ability as well as the localization of TRIOBP to the cellular actin cytoskeleton in the stereocilia rootlets (Bao et al., 2013). Since DFNB28 is a recessive condition, it would imply that a single intact R1 motif would be sufficient for normal hearing.

Individual IV.9 in Family TS005 carries the heterozygous c.572delC p.Pro191Argfs*50 variant and has normal hearing. This individual retains a single, complete R1 repeat motif, but unlike the deaf family members, also retains an intact R2 repeat motif. Although half of the TRIOBP protein produced in this individual will lack both R1 and R2 repeat motifs *in cis*, the other half will be normal. The grandmother, individual II.2, carries the truncating c.3510_3513dup p.Pro1172Cysfs*13 mutation situated inside the R2 motif (Figure 3). This means that half of her TRIOBP produced will be truncated within the R2 repeat motif while the other half is unaffected. She too has normal hearing. The deceased grandfather, individual II.1 in Family TS005, presumed to have carried the heterozygous c.572delC p.Pro191Argfs*50 variant, would have produced 50% intact and 50% truncated TRIOBP without R1 and R2.

Taken together, the results suggest that truncation of TRIOBP within repeat motif R2 is sufficient to cause hearing loss despite R1 being intact. Thus, while a single intact R1 motif is adequate for actin‐binding and bundling in the stereocilia rootlets, a truncated R2 *in cis*, prevents TRIOBP from being fully functional and maintaining physiological hearing.

The control family TS067 in whom compound heterozygous missense TRIOBP variants of unknown significance were observed was from the same population group and geographical region. In this family, the genotype did not segregate with the deafness phenotype, with compound heterozygous variants c.2532G > T p.Leu844Arg, c.2590C > A p.Asn867Lys, c.3484C > T p.Pro1161Leu, and c.3621 T > C p.Phe1187Leu occurring among the hearing impaired as well as the normal hearing individuals in varying combinations (Figure 2). Whereas individual II1, who is profoundly deaf, carries all four variants, his normal hearing sister has three of the variants, while both parents have two variants each, meaning that these variants are unlikely to be associated with his hearing phenotype. He also has variants in two other known deafness genes which are still under investigation. The gene responsible for deafness in this individual remains to be determined. Family TS005 also had two additional missense variants c.505C > A p.Asp168Glu and c.3636 T > A p.Leu1212Gln. Individual IV.9 who has normal hearing carries variants c.505C > A p.Asp168Glu and c.572delC p.Pro191Argfs*50. Although p.Pro191Argfs*50 truncates half of her TRIOBP protein losing both R1 and R2, the other half retains intact R1 and R2 repeat motifs. Despite the missense variant p.Asp168Glu, she retains normal hearing function. We, therefore, classified variant p.Asp168Glu as benign. A cousin to the proband who carries variant p.Leu1212Gln also has normal hearing. We consider this variant p.Leu1212Gln as likely benign. From these findings, we infer that although variant p.Pro1161Leu occurs inside the R2 repeat motif, this nontruncating change is unlikely to interfere with the functioning of the R1 repeat motif *in cis*.

Analysis of the previously reported TRIOBP variants in the literature as well as those from the current study implies that truncating variants involving exons 6 and 7 segregate with deafness and are therefore correctly classified as pathogenic mutations (Tables 1 and 2, Figure 3). All pathogenic variants truncate TRIOBP such that repeat motifs R1 and/or R2 are (partly) lost. By contrast, nontruncating variants in the same exons, including those located within repeat motifs R1 and R2, do not segregate with deafness (Table 3, Figure 3). Most heterozygous missense variants in exon 7 are thus likely to be tolerated. Missense variants in exons upstream or downstream of exon 7 will thus also likely be tolerated and not cause hearing loss (Table 3, Figure 3) implying that the published variants classified as of “uncertain significance” are likely benign.

**TABLE 1:** Published characterized TRIOBP variants in resolved DFNB28 families

Genotype				Phenotype	Ethnicity	Reference
Allele 1 code	Allele 1	Allele 2 code	Allele 2	Pathogenicity of variants		
**1A1**	c.572del (p.Pro191Argfs*50)	**1A2**	c.3510_3513dupTGCA (p.Pro1172Cysfs*13)	**Pathogenic**	**South African**	**Current study** Yan et al. (2016)
	**c.2532G > T (p.Leu844Arg)**		**c.3484C > T (p.Pro1161Leu)**	**Benign**	**South African**	**Current study**
	**c.2590C > A (p.Asn867Lys)**		**c.3621 T > C (p.Phe1187Leu)**	**Benign**	**South African**	**Current study**
	**c.505C > A (p.Asp168Glu)**		c.572del (p.Pro191Argfs*50)	**p.Asp168Glu Benign**	**South African**	**Current study**
1B1	c.889C > T (p.Gln297*)	1B2	c.889C > T (p.Gln297*)	Pathogenic	Indian	Bosman (1989); Riazuddin et al. (2006)
1C1	c.1039C > T (p.Arg347*)	1C2	c.1039C > T (p.Arg347*)	Pathogenic	Palestinian	de Monvel and Petit (2010); Shahin et al. (2006)
1D1	c.1039C > T (p.Arg347*)	1D2	c.1741C > T (p.Gln581*)	Pathogenic	Palestinian	de Monvel and Petit (2010); Shahin et al. (2006)
1E1	c.1039C > T (p.Arg347*)	1E2	c.3055G > A (p.Gly1019Arg)	Pathogenic	Palestinian	de Monvel and Petit (2010); Shahin et al. (2006)
1F1	c.1741C > T (p.Gln581*)	1F2	c.1741C > T (p.Gln581*)	Pathogenic	Palestinian	de Monvel and Petit (2010); Shahin et al. (2006)
1G1	c.2355_2356del (p.Arg785Serfs*50)	1G2	c.2355_2356del (p.Arg785Serfs*50)	Pathogenic	Turkish	Diaz‐Horta et al. (2012)
1H1	c.2362C > T (p.Arg788*)	1H2	c.2362C > T (p.Arg788*)	Pathogenic	Turkish	Bosman (1989); Riazuddin et al. (2006)
1 J1	c.2653del (p.Arg885Alafs*120)	1 J2	c.5014G > T (p.Gly1672*)	Pathogenic	Dutch	Wesdorp et al. (2017)
1 K1	c.2968C > T (p.Arg990*)	1 K2	c.2968C > T (p.Arg990*)	Pathogenic	Pakistani	Gu et al. (2015); Naz et al. (2017)
1 L1	c.3202C > T (p.Arg1068*)	1 L2	c. 3202C > T (p.Arg1068*)	Pathogenic	Pakistani	Bosman (1989); Riazuddin et al. (2006)
1 M1	c. 3202_3203del (p.Asp1069Cysfs*14)	1 M2	c.3202_3203del (p.Asp1069Cysfs*14)	Pathogenic	Indian	Bosman (1989); Riazuddin et al. (2006)
1 N1	c.3232dup (p.Arg1078Profs*6)	1 N2	c.3232dup (p.Arg1078Profs*6)	Pathogenic	Indian	Bosman (1989); Riazuddin et al. (2006)
1P1	c.3232dup (p.Arg1078Profs*6)	1P2	c.3460_3461del (p.Leu1154Alafs*29)	Pathogenic	Dutch	Wesdorp et al. (2017)
1Q1	c.3349C > T (p.Arg1117*)	1Q2	c. 3349C > T (p.Arg1117*)	Pathogenic	Indian	Bosman (1989); Riazuddin et al. (2006)

*Note*: NCBI Reference Sequence: NC_000022.11 used.

**TABLE 2:** Published pathogenic TRIOBP variants among DFNB28 families without segregation data

Genotype				Phenotype	Ethnicity	Reference
Allele 1 Code	Allele 1	Allele 2 Code	Allele 2	Pathogenicity of variants		
2A1	c.1420C > T (p.Arg474*)	2A2	c.1420C > T (p.Arg474*)	Pathogenic	Unknown	Howard and Ashmore (1986); Kitajiri et al. (2010)
2B1	c.1567C > T (p.Arg523*)	2B2	c.1567C > T (p.Arg523*)	Pathogenic	Unknown	Howard and Ashmore (1986); Kitajiri et al. (2010)
2C1	c.2218C > T (p.Gln740*)	2C2	c.2218C > T (p.Gln740*)	Pathogenic	Unknown	Howard and Ashmore (1986); Kitajiri et al. (2010)
2D1	c.2521C > T (p.Arg841*)	2D2	c.2521C > T (p.Arg841*)	Pathogenic	Turkey	Yan et al. (2016); Furness et al. (2008); unpublished data±
2E1	c.2581C > T (p.Arg861*)	2E2	c.2758C > T (p.Arg920*)	Pathogenic	Chinese	Fardaei et al. (2015); Gu et al. (2015)
2F1	c.2581C > T (p.Arg861*)	2F2	c.3089del (p.Pro1030Leufs*183)	Pathogenic	American	Furness et al. (2008); Yan et al. (2016)
2G2	c.3466G > T (p.Glu1156*)	2G2	c.3466G > T (p.Glu1156*)	Pathogenic	Unknown	Howard and Ashmore (1986); Kitajiri et al. (2010)

**TABLE 3:** Published uncharacterized TRIOBP variants in comparison with the South African missense variants

Genotype				Phenotype	Ethnicity	Reference
Allele 1 code	Allele 1	Allele 2 code	Allele 2	Pathogenicity of variants		
3A1	**c.2532G > T (p.Leu844Arg)**	3A2	**c.3484C > T (p.Pro1161Leu)**	**Benign**	**South African**	**Current study**
3B1	**c.2590C > A (p.Asn867Lys)**	3B2	**c.3621 T > C (p.Phe1187Leu)**	**Benign**	**South African**	**Current study**
3C1	**c.505C > A (p.Asp168Glu)**	3C2	c.572del (p.Pro191Argfs*50)	**p.Asp168Glu Benign**	**South African**	**Current study**
3D1	**c.3636 T > A p.Leu1212Gln**		**Unknown**	**Likely benign**	**South African**	**Current study**
3E1	c.154G > A (p.Asp52Asn)	3E2	Unknown	Uncertain significance	Japanese	Miyagawa et al. (2013); Yan et al. (2016)
3F1	c.2992G > A(p.Ala998Thr)	3F2	c.5767G > A(p.Ala1923Thr)	Uncertain significance	Unknown	Sloan‐Heggen et al. (2016); Zhao and Muller (2015)
3G1	c. 3349C > T (p.Arg1117*)	3G2	c.4691G > C (p.Gly1564Ala)	Uncertain significance	Unknown	Sloan‐Heggen et al. (2016); Zhao and Muller (2015)
3H1	c.3451A > G (p.Met1151Val)	3H2	c.4187C > G (p.Pro1396Arg)	Uncertain significance	Unknown	Fardaei et al. (2015); (2015)
3 J1	c.3662G > A (p.Arg1221Gln) c.3942G > C (p.Glu1314Asp)	3 J2	c.6736G > A (p.Glu2246Lys)	Uncertain significance	Unknown	Sloan‐Heggen et al. (2016); Zhao and Muller (2015)
3 K1	c.4840G > T (p.Gly1614Cys)	3 K2	Unknown	Uncertain significance	Japanese	Miyagawa et al. (2013); Yan et al. (2016)
3 L1	c.5519G > A (p.Arg1840His)	3 L2	Unknown	Uncertain significance	Japanese	Miyagawa et al. (2013); Yan et al. (2016)
3 M1	c.6362C > T (p.Ser2121Leu)	3 M2	c.6362C > T (p.Ser2121Leu)	Uncertain significance	Iranian	Fardaei et al. (2015); Flock and Cheung (1977)
3 N1	c.6860G > A (p.Arg2287His)	3 N2	Unknown	Uncertain significance	Japanese	Miyagawa et al. (2013); Yan et al. (2016)

*Note*: NCBI Reference Sequence: NC_000022.11 used.

One study reported pathogenic TRIOBP mutations in two isolated Dutch individuals with congenital moderate hearing loss (Wesdorp et al., 2017), c.2653del p.Arg885Alafs*120 (1 J1) and c.5014G > T p.Gly1672* (1 J2). While 1J1 incurs a loss of R2, 1J2 is far downstream of repeat motifs R1 and R2 (Figure 3). As noted by the authors, variant c.5014G > T p.Gly1672* is an outlier as it would be the first identified pathogenic variant in DFNB28 that does not affect isoform class TRIOBP‐4. A single copy of *TRIOBP* encoding wild‐type TRIOBP‐4 would be insufficient for normal hearing, requiring at least one copy of *TRIOBP*‐encoding TRIOBP‐5 for normal inner ear function. Based on Figure 3, although p.Gly1672* is a truncating variant, it is too far downstream to affect the intact R1 or R2 repeat motifs *in cis*.

## 5 CONCLUSION

Bao et al. (2013) demonstrated in vitro that repeat motif R1 is the major actin‐binding domain of TRIOBP in cochlear hair cells. Our analysis of the published TRIOBP variants and the variants in the South African families in the current study has shown that genotypes combining two truncating variants affecting repeat motifs R1 and/or R2 lead to a deafness phenotype, while a truncating variant affecting repeat motifs R1 and/or R2 combined with a missense variant does not. Homozygous truncating variants affecting repeat motif R2 cosegregate with the deafness phenotype. The truncation of R2 repeat motifs in both alleles thus leads to a deafness phenotype by affecting the physiological role of TRIOBP by as yet unknown mechanisms. Normal hearing by contrast requires the presence of at least one mostly intact R2 repeat motif *in cis* with the normal R1 repeat motif.

Our results also suggest that TRIOBP‐truncating variants in the C‐Terminal downstream of the repeat motifs R2 and of exon 7 are unlikely to interfere with the actin‐binding and bundling activities of the repeat motifs and are therefore unlikely to lead to a deafness phenotype. Published frameshift and/or truncating mutations involving exons 6 and 7 tend to segregate with the deafness phenotype, while missense variants in the same exons do not.

Further research, such as protein assays and functional studies in mutant animal models, is needed to confirm these hypotheses.

## FUNDING INFORMATION

This study was supported by the Fulbright Senior Research Scholar Award and the University of Pretoria RDP funding to RI Kabahuma; R01 DC05575 and R01 DC012115 from the National Institutes of Health/National Institute on Deafness and Other Communication Disorders to Xue Zhong Liu; the South African Medical Research Council and the University of Pretoria to MS Pepper.

## CONFLICT OF INTEREST

The authors declare no conflict of interest.

## ETHICAL STATEMENT

This study was approved by the Research Ethics Committee of the Faculty of Health Sciences, University of Pretoria, South Africa, Ethics Approval number 395/2014, and the Institutional Review Board of the University of Miami, Miller School of Medicine (USA). Signed informed consent was obtained from each participant or, in the case of a minor, from the parents.

## Data Availability

The data that support the findings of this study are available from the corresponding author upon reasonable request.
